# Improvement and Validation of a Multi-Locus Variable Number of Tandem Repeats Analysis (MLVA8+) for *Klebsiella pneumoniae*, *Klebsiella variicola*, and *Klebsiella quasipneumoniae*

**DOI:** 10.3390/microorganisms11020444

**Published:** 2023-02-10

**Authors:** Deyan Donchev, Ivan N. Ivanov, Ivan Stoikov, Stefana Sabtcheva, Yordan Kalchev, Marianna Murdjeva, Elina Dobreva, Rumyana Hristova

**Affiliations:** 1 National Reference Laboratory for Control and Monitoring of Antimicrobial Resistance, Department of Microbiology, National Center of Infectious and Parasitic Diseases, 26 Yanko Sakazov Blvd., 1504 Sofia, Bulgaria; 2 Laboratory for Clinical Microbiology, National Oncology Center, 6 Plovdivsko pole Str., 1797 Sofia, Bulgaria; 3 Department of Medical Microbiology and Immunology, “Prof. Dr. Elissay Yanev”, Medical University—Plovdiv, 15-A Vasil Aprilov Blvd., 4002 Plovdiv, Bulgaria

**Keywords:** MLVA, MLST, Klebsiella, pneumoniae, variicola, typing, VNTR, WGS

## Abstract

The genotyping of the multidrug-resistant *Klebsiella pneumoniae* species complex is essential to identify outbreaks and to track their source and spread. The aim of this study was to improve and extend the typeability, availability, cost and time efficiency of an existing multi-locus VNTR analysis (MLVA). A modified scheme (MLVA8+) was adopted and validated for strain-level differentiation of the three *Klebsiella* species involved in human pathology. A diverse set of 465 *K. pneumoniae* clinical isolates from 22 hospitals and 3 outpatient laboratories in Bulgaria were studied, where 315 were carbapenem-resistant. The MLVA8+ typeability was significantly improved and the typing data were validated against 158 isolates which were previously typed by WGS. The MLVA8+ results were highly concordant with the classic 7-locus MLST and the novel *K. variicola* MLST, but had greater congruency coefficients (adjusted Wallace). A major advantage was the differentiation of the hybrid cluster ST258 into its corresponding clades. Furthermore, the applicability of MLVA8+ was demonstrated by conducting a retrospective investigation of the intra-hospital spread of *bla*KPC-, *bla*NDM- and *bla*OXA-48-like producers. The MLVA8+ has improved utility and extended typing scope to *K. variicola* and *K. quasipneumoniae*, while its cost and time-to-result were reduced.

## 1. Introduction

*Klebsiella pneumoniae* (KPn) has been increasingly recognized as a ubiquitous causative agent of infectious diseases in both the health-care sector and in the community [1,2]. It accounts for nearly one-third of all Gram-negative urinary tract infections, pneumonia, surgical site infections, bacteremia [3,4] and neonatal sepsis [5] and is therefore perceived by the WHO as a critical public health threat [6].

KPn have been strongly associated with acquired resistance against aminoglycosides, third generation cephalosporins, last line carbapenems and colistin [3,7,8]. According to a recent systematic review, the pooled prevalence of nosocomial multidrug-resistant KPn infections was estimated to reach 32.8% [9]. Evidence has shown that the propensity of KPn isolates to spread among hospital settings correlates with the degree of resistance, whereas the carbapenemase carrying isolates are the most transmittable [10]. In addition, the dynamic taxonomy [11], the inability of traditional and recent technologies for precise identification, and the virulence and resistance plasticity within *Klebsiella pneumoniae* species complex (KpSC) [12,13] necessitate the need for accurate and reliable strain-level differentiation.

In this regard, great effort has been devoted to developing molecular epidemiological tools for typing and distinguishing beyond species level for the purpose of tracking transmission routes of KPn isolates and to predict their relatedness. A variety of useful techniques and methods for KPn typing, such as capsular typing (K- and O- typing), pulsed-field gel electrophoresis, multi-locus sequence typing (MLST) and, more recently, whole-genome sequencing (WGS) have been widely used over the years, each having distinct advantages and applicability. However, all of them are either laborious, expensive or require specific equipment or expertise to interpret the results, hence the necessity of a more straightforward typing approach.

Similarly, tandemly repeated sequences within bacterial genomes are utilized for bacterial typing as another source of genetic polymorphism. Repeated sequences are products of randomly occurring slipped strand mispairing mutation that can result in either addition or deletion of repeats, depending on different loci or alleles [14]. Evaluating the variable number of tandem repeats (VNTR) at multiple loci is known as multi-locus VNTR analysis (MLVA) and has been used as a genotyping method providing insights into bacterial population structure [15].

MLVA typing schemes were successfully developed and used for typing of *Staphylococcus lugdonensis* [16], *Staphylococcus aureus* [17], *Bacillus anthracis* [18], *Brucella spp.* [19], *Shigella* and *Escherichia coli* [20]. For KPn, an eight loci MLVA scheme was described previously [21], where fluorescently labeled primers were used to amplify selected VNTR loci in one PCR reaction and differentiate them based on size and color. Although the proposed design shared similar typing resolution with the MLST method that is perceived as gold standard, it required Sanger sequencer with costly fluorescently labeled primers and additional reagents for fragment size differentiation (e.g., fluorescent DNA ladders). Another drawback is the relatively large turnaround time (at least 48 h) that might be crucial in outbreak situations.

Herein, we present a modified and improved version of this method (MLVA8+), that is at least as discriminatory as MLST, more cost-effective, rapid and requires a thermocycler and a capillary gel electrophoresis system. It was used to investigate a large heterogenic sample set of 464 KPn isolates collected from Bulgarian hospitals between January 2012 and June 2021. In addition, the method was further tested in silico with a sample set of *K. variicola* (KVa) genomes against a novel MLST scheme from 2019.

## 2. Materials and Methods

### 2.1. Bacterial Strains

Three distinct sets of KpSC strains were used to test the modified typing version MLVA8+. The first sample set (set A) consisted of 464 clinical strains from 22 hospitals and 3 outpatient private laboratories from all six NUTS-2 regions in Bulgaria. Of these strains, 298 were clinical strains collected by the National Reference Laboratory for Control and Monitoring of Antimicrobial Resistance at the National Center for Infectious and Parasitic Diseases, Bulgaria, through routine surveillance from January 2012 to December 2021. In total, 315/464 were carbapenemase producers. The second one (set B) consisted of 182 clinical isolates with known sequence types (ST). Of these, 158 strains originated from 11 Bulgarian hospitals, previously submitted for WGS on Illumina Systems with (2 × 150 bp) through the ECDC survey of carbapenem- and/or colistin-resistant *Enterobacterales* (https://www.ecdc.europa.eu/en/publications-data/ecdc-study-protocol-genomic-based-surveillance-carbapenem-resistant-andor, accessed on 7 February 2023). From the remaining 24 strains, 10 were sequenced locally by preparing libraries with Illumina DNA Prep kit and sequenced on MiSeq System with V3 (2 × 300 bp cycles), (Illumina, Inc., San Diego, CA, USA), whereas the remaining 14 strains were typed by classical MLST. The third sample set (set C) consisted of 88 KVa genomes acquired from NCBI database. Finally, two reference strains KPn ATCC_13883 (ASM74213v1) and KQpn ATCC_700603 (CP014696) were also included. Complete strain information is included in Supplementary Tables S5–S7. We developed a specific coding convention (Supplementary File S1) and proposed slight modifications to the scheme and the conversion table from the original scheme.

### 2.2. MLVA Design

A published eight loci single-tube MLVA typing scheme developed by Brink A. et al. was adopted for the design [10]. Based on the meticulously selected loci we created a simpler and cost-effective design yielding corresponding results in less hands-on-time, while expanding its typing scope. First, we studied in silico all possible variations in the repeat copy number and in amplicon size ranges associated with each VNTR locus in KPn, KVa and KQpn genomes available in the NCBI up to December 2019. New sets of multiplex primers were designed with Geneious R10.2 (https://www.geneious.com, accessed on 6 December 2022, quality checked with Oligo 7 [22] and were further grouped in two multiplex PCRs (MLVA8.1 and MLVA8.2) so that each expected amplicon within the same reaction does not overlap with the others by size. The primer pairs were deliberately chosen to target VNTR loci with shorter repeat units in order to improve the resolution of capillary gel electrophoresis analysis. KPn ATCC_13883 (ASM74213v1), MGH 78578 (GCA_022305115.1) and KQpn ATCC 700603 (CP014696) were used as reference genomes. Primer sequences and PCR setup information are available on Supplementary Tables S1 and S2.

### 2.3. DNA Extraction and Multiplex PCR

**Figure 1:**
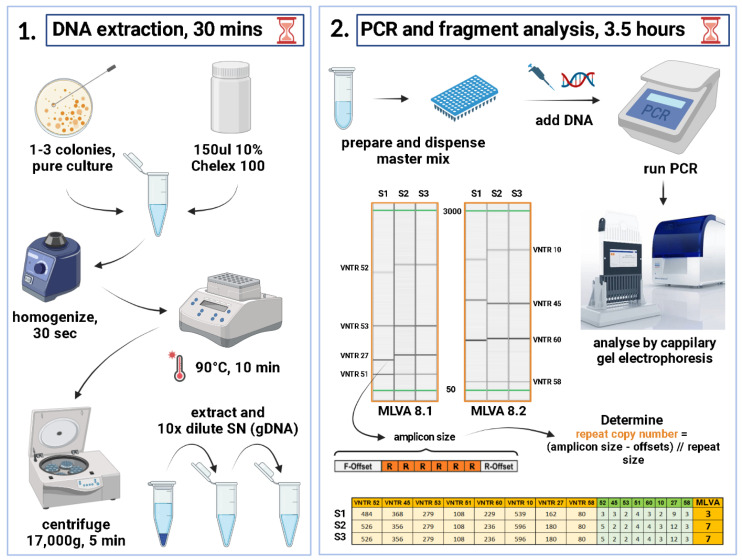
A schematic representation of the MLVA+ protocol. The full protocol requires a minimum of 4 h from 18–24 h pure cultures to generate genotypes. This figure was created with BioRender (https://biorender.com/, accessed 10 January 2022).

Strains were cultured overnight in Columbia or Muller-Hinton agar and 2–3 colonies were suspended in 150 µL 10% Chelex 100 in TE buffer (Bio-rad), vigorously homogenized for 30 sec and incubated at 96 °C for 10 min. The tubes were rapidly cooled on ice for 2 min and centrifuged at 17,000× *g* for 5 min. The supernatant was transferred and diluted to 5–10 ng/µL in TE buffer or stored undiluted at −20 °C (Figure 1). DNA was added to each of MLVA8.1 and MLVA8.2 mixes containing PCR buffer, PerpetualTaq (EurX, Poland), Tetraethyl ammonium chloride, Dimethyl sulfoxide, dNTPs and corresponding primer sets and subjected to amplification (Supplementary Table S2). High-resolution capillary electrophoresis (QIAxcel, QIAGEN) was used with protocol 0M800 (3 kV for 800 s) for fragment analysis. Control DNA from strains with known VNTR copy numbers were loaded alongside with samples for accurate size identification and inter-run normalization.

### 2.4. VNTR Interpretation, Genotype Assignment and Phylogenetic Relatedness

**Figure 2:**
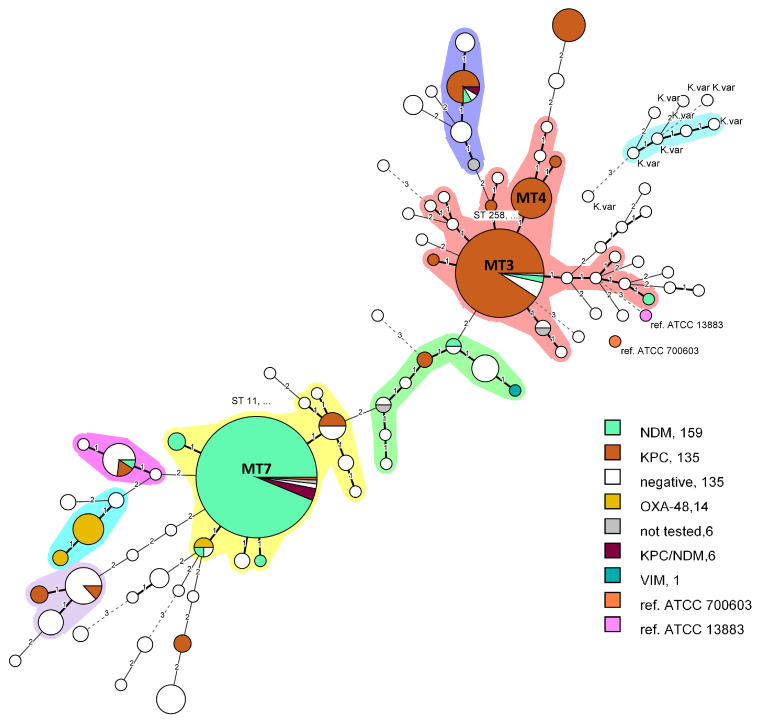
Minimum spanning tree, created with BIONUMERICS v8.1.0 from *n* = 466 isolates. Bolded branches represent difference in one VNTR locus, thin branches—two VNTRs difference and dashed lines—difference in three or more VNTR loci. The differences in three or more loci were considered not related. Clonal complexes were defined by grouping together neighboring MLVA types that differed in only one VNTR (Single Locus Variant, SLV), consisted of at least three nodes and four isolates. Nodes represent a pie chart proportion of the encoded carbapenemases.

The obtained amplicon sizes were converted to VNTR copy numbers by first subtracting the offset sequences flanking the repeat region and the remaining sequence divided by the repeat size (Figure 1). Repeat copy numbers ranged from zero to the highest hypothetical number. In case of non-integer value, the formula rounded up to the closer integer value. The genotyping was carried out by creating a specific Excel spreadsheet (Supplementary Table S8) with formulas (Figure 1) that converts the amplicon sizes of each VNTR into repeat copy number, therefore creating an MLVA profile for each isolate, then manually assigning an MLVA type (MT) starting from 1 and next number was assigned to each novel profile. A dedicated *Klebsiella* database was created in MLVA Bank (https://microbesgenotyping.i2bc.paris-saclay.fr/databases/public, accessed on 3 February 2023) for MT assignment. For instance, the reference strain ATCC 13883 happened to be MT 66 with MLVA profile 4-3-0-4-4-2-5-4, i.e., numbers of repeats within VNTRs 52-45-53-51-60-10-27-58. The obtained MTs were imported into BIONUMERICS software v8.1 and a Minimum Spanning Tree (MST) was produced for visualizing closely related isolates (Figure 2). The discriminatory power was calculated using Simpson’s diversity index (DI) with online tool (http://www.comparingpartitions.info/?link=Tool, accessed on 2 February 2023) [23].

### 2.5. Validation and Comparison to MLST

**Figure 3:**
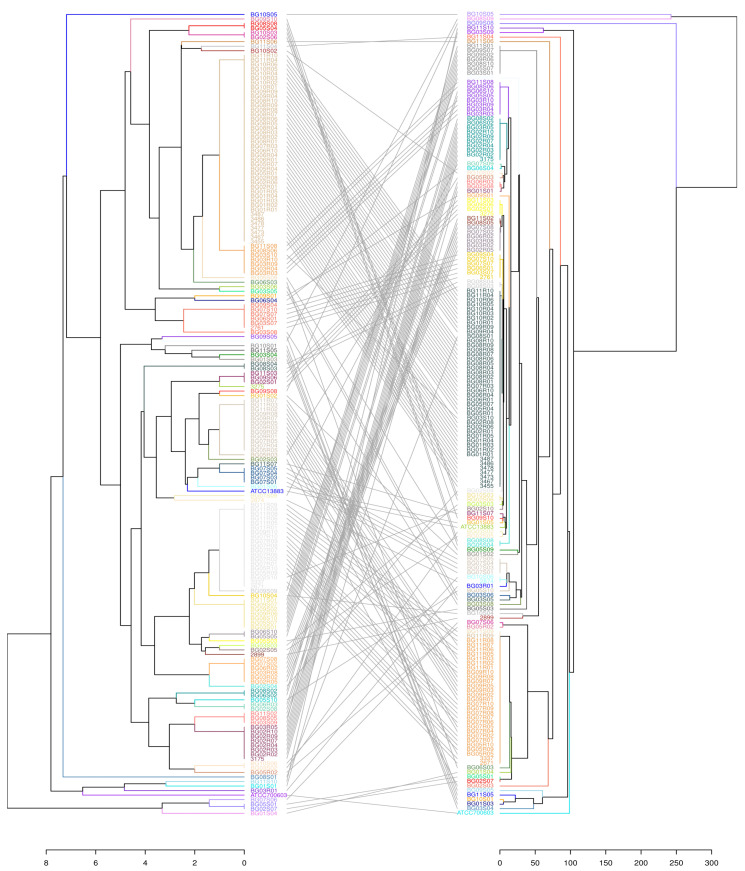
A tanglegram depicting the overall congruence between the modified MLVA8 (**left**) and the gold standard MLST (**right**) on set B. Lines connect the corresponding isolates in the different trees. Both allele tables were imported into R studio environment. Distance matrices were created with *dist* function (“*euclidean*” for MLVA, “*manhattan*” for MLST), hierarchically clustered with *hclust* function using UPGMA method and represented as dendrograms. Finally, R package dendextend v1.16.0 was used to compare both phylogenetic trees and connect the corresponding clusters. The predominantly parallel lines illustrate the significant correspondence of MLVA types with the specific STs.

The strain set A (*n* = 464) was used for validation purposes. The raw NGS data (project strains included) was fed into the Unicycler assembly tool v0.4.8 for de novo assembly with default settings [24]. Next, an in silico PCR tool (https://github.com/egonozer/in_silico_pcr, accessed on 6 December 2022) with max length parameter of 1000 bp and the newly designed primers was utilized to perform in silico MLVA8+ on the obtained draft genomes (script available here: https://github.com/maddne/In-silico-PCR-for-Kpn-MLVA8Plus.git, accessed on 10 January 2022). The MLST analysis was performed with the mlst tool v.2.23.0 tool (https://github.com/tseemann/mlst, accessed on 1 December 2022) [25]. The overall congruency was visualized in tanglegram (Figure 3) created with R [26] and package dendextend v1.16.0. Simpson’s DI and adjusted Wallace’s index (AWI) were calculated [27].

### 2.6. Comparison of MLVA8+ with K. variicola Specific MLST

**Figure 4:**
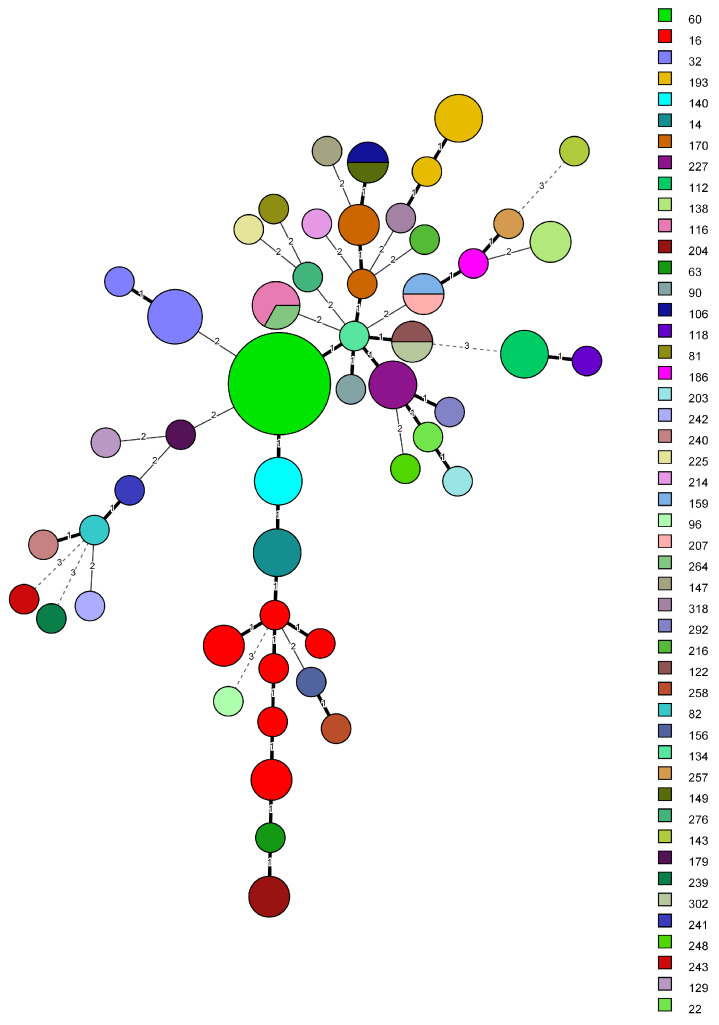
Minimum spanning tree, created with BIONUMERICS v8.1.0 from 88 *K. variicola* isolates. Bolded branches represent one VNTR difference, thin branches—two VNTR difference—and dashed lines—difference in three or more VNTR loci. Difference in three or more loci were considered not related. Sequence types are represented in different colors and in the legend. Grouping of non-identical STs into a single MLVA types occurred four times.

A newly developed MLST typing scheme for KVa was introduced in 2019 [28]. For the purpose of comparing both schemes, we downloaded all publicly available KVa genomes (*n* = 650) from NCBI using the tool ncbi-genome-download v.0.3.1 (https://github.com/kblin/ncbi-genome-download, accessed on 10 November 2022) using assembly level parameters and specifying complete, chromosome and scaffold. Next, we downloaded known alleles, primers and sequence type table from (https://mlstkv.insp.mx/, accessed on 6 January 2023) and created a custom MLST typing scheme within the mlst tool [25]. Existing STs were yielded for only 90/650 genomes and those were subjected to in silico pcr as explained in section “Validation and comparison to MLST”. For a small fraction of all VNTR loci, manual allele searching was necessary. For this part, the motif search package in Geneious Prime^®^ 1 January 2021 was used. Finally, 88/90 genomes were in silico genotyped, Supplementary Table S7. The correspondence between both typing methods was calculated with aWI [27] and depicted in Figure 4.

### 2.7. MLVA Epidemiological Study in a Single Hospital

**Figure 5:**
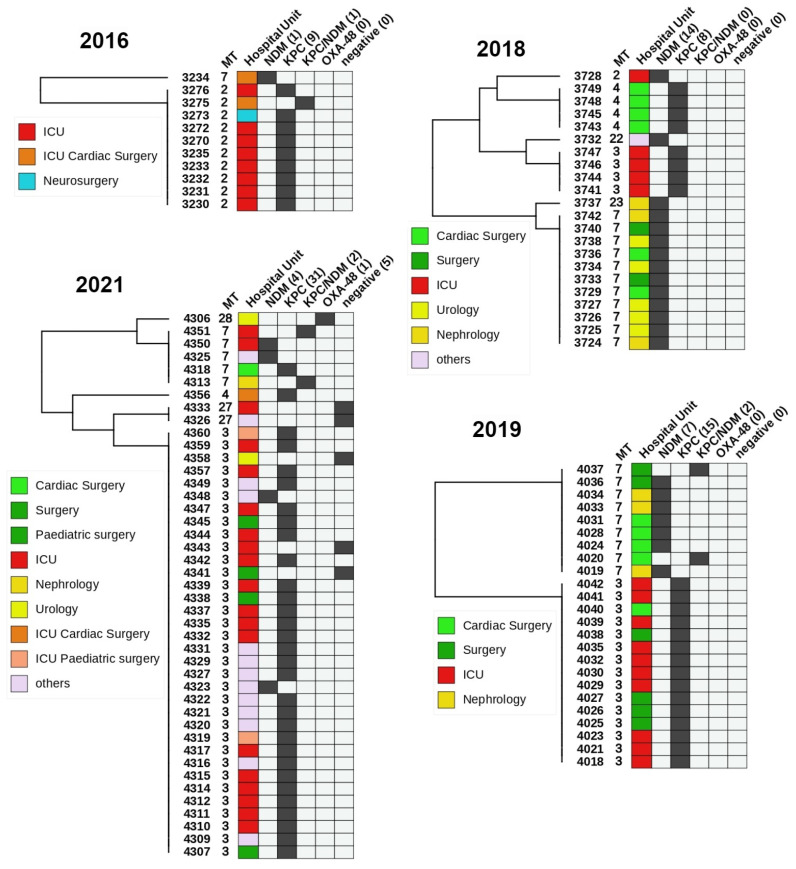
A heatmap representation of carbapenemase producing *K. pneumoniae* from a single hospital over the period from 2016 to 2021. The left side is an UPGMA clustered tree with leaves labelled with strain IDs. The heatmap includes columns with MLVA type (MT), Hospital unit, NDM, KPC, NDM + KPC, and OXA-48. Positive results are colored in black, whereas negative is light grey. The most notable results were the prevalence of KPC-producing isolates in the ICU and NDM-producing isolates in the Urology and Nephrology. Both KPC and NDM isolates were isolated from various surgery units almost equally.

To validate the application of MLVA for molecular epidemiology we filtered out all strains from hospital A from set A. We selected the hospital based on the highest number of isolates. The subset included 100 strains (without strains from set B) isolated in four years 2016, 2018, 2019 and 2021. For each year, UPGMA dendrograms were clustered with R. Metadata, such as sample ID, MTs, hospital wards and carbapenemase types, which were represented in the form of a heatmap (Figure 5). The dendrogram and heatmaps were displayed and annotated with iTOL v5 [29].

## 3. Results

### 3.1. In-Silico Analysis

First, the eight VNTR loci previously proposed by Brink et al. have been re-analyzed and compared across the reference genomes [21]. It has become evident that all loci were present among KPn, KQpn and KVa, but the target sites of the published primers were either lacking or significantly altered in KQpn and KVa and are therefore unusable (data not shown). A few changes in the repeat descriptions were also necessary. For example, the repeat sequence GCA for VNTR 58 was changed to CAG after thorough testing and manually inspecting the behavior of the VNTR among other genomes. Consequently, the number of assigned repeat copy range changed from (0–10) to (2–12). Regardless, the amplicons continued to vary by 3 bp. Similar observation was made for VNTR 10, where genomes with more than the suggested maximum of four repeats were found in NCBI. The VNTR52 repeat size was 21 bp; however, repeat copy numbers of 4, 5 and 6 in some genomes would generate varying PCR product sizes from 500 to 505, 521 to 525 and 542 to 546 bp, respectively, and were taken into consideration. In addition to this analysis, we decided to simplify and streamline the process by generating the amplicons in a generic multiplex PCR. Therefore, we constructed a theoretical multiplex PCR amplicon pattern, where none of the amplicons would have overlap with the adjacent ones. This task has been accomplished entirely in silico with the information from publicly available genomes. Finally, new primer pairs were designed and quality checked for homo- and heterodimer formation and thermodynamic efficiencies (Supplementary Table S1).

### 3.2. Comparison to MLST Methods

The modified typing method was validated on an extensive diverse sample set. In the set A (*n* = 464), the discriminatory power was 0.846 (CI 95% 0.819–0.873). The Simpson’s index may vary depending on the sample heterogeneity. There are 158 identical strains MLVA 7 (ST11), a clonal group that is widespread in Bulgaria [30,31,32,33]. In addition, 103/464 strains are MLVA 3 and 4 (ST258), which is the second most spread clonal group in the country [34]. The highest Dis were found in VNTRs 27 (67%), 52 (64.2%), 60 (64.7%) and 10 (65.6%), whereas the lowest diversity of 0.9% was found in VNTR 51 (Supplementary Table S5). Negative VNTR values were observed for two strains BG08S01Kpn (VNTR 53) and BG10S05Kpn (VNTR 51), from set B (*n* = 182) indicating less repeats that the minimum expected (0 repeats). The in vitro typeability increased to 99.78% (1 strain, not included) from 92.85% in the original MLVA.

In order to validate the method and compare it to MLST, we used a sample subset B with known STs. In total, 64 distinct MLVA types were identified versus 60 STs. The MVLA DI for was 0.926 (0.900–0.951), whereas for MLST it stood at 0.907 (0.879–0.935). MLVA was able to distinguish more precisely isolates with identical STs. A major result was the statistically significant differentiation (*p* < 0,05) of the hybrid cluster ST258 into two MLVA sub-clusters 4 (12 strains) and 3 (18 strains), allowing for more accurate tracking of genetic clades evolution. Next, ST340 (8 strains) was split into two MTs 86 (2) and 8 (6) and two ST11 were redistributed into MLVA types 8 and 84, with the latter forming a distinct monophyletic clone. In general, identical ST isolates were only sporadically separated into newly formed MTs, hence the higher number of unique MLVA types. On the other hand, there were four instances where varying STs were also grouped together into a single MT (ST116/264 => MT130), (ST149/106 => MT110), (ST207/159 => MT95) and (ST122/302 => MT133). However, in those occurrences merged STs were represented by a single isolate each. To represent the probability of such events, an aWI as a congruence measure between MLVA and MLST was calculated. It was estimated that if two strains were in the same MT there is 97.6% chance of being the same ST (CI 0.947–1.000), while conversely it was 76.4% (CI 0.681–0.846). This was primarily due to the separation of ST258. For both schemes KVa strains were not merged with KPn strains. A correlation between phylogenetic trees is shown in Figure 2.

The same analysis was performed against the KVa newly available MLST scheme [28]. Initially, 650 KVa genomes were downloaded for comparison; however, only 88 genomes were successfully assigned both complete ST and MT. The remaining ones were discarded due to matching either novel alleles or partially to known alleles in the MLST scheme. Strain distribution was comparable for both schemes, 52 MTs versus 48 STs. Although both share virtually similar Simpson’s DI 0.968 (0.946–0.990) for MLVA versus 0.960 (0.938–0.982) for MLST, the aWI showed that there is a 95.8% chance isolates sharing the same MT to be of identical ST compared to only 76.4% (CI 0.712–0.815) for MLST, reflecting the higher discriminatory capacity of MLVA over MLST. One drawback was found when comparing the results that could explain the congruency gap. From the eight ST16 KVa genomes six received unique MTs, effectively separating them into separate clusters. Notably, the differences were mostly due to VNTR 58 and to a lesser extent to VNTR 52. Two of those MLVA types (100, 131) had two genomes each, reducing the likelihood of an erroneous allele assignment. Similar to KPn MLST, on a few occasions, monophyletic STs were grouped together into one MTs. Moreover, no KVa strains from this set were assigned a MT that includes KPn or KVa strains from the Bulgarian strains (sets A and B). All the information is available in Supplementary Tables S5–S7. Sequence type distribution among MLVA clusters are differently colored in Figure 4.

### 3.3. Phylogenetic Relatedness and Carbapenemases

Carbapanemases of types NDM, KPC, OXA-48-like and VIM produced by strains from set A (*n* = 464) were clustered in an MST and visualized in different colors on Figure 2. Two major clonal complexes were formed. The first one (pink) included the MT3 and MT4 nodes which differed only in one VNTR locus and 92.2% of them carried *blaKPC*. Both nodes corresponded to ST258. In addition, three more single isolate nodes encoding KPC were directly connected to MT3 and four indicating the clonal relatedness. Within MT3 node only two strains were negative for carpanemases and two carried *blaNDM.* The other major clonal complex (yellow) was formed by MLVA 7 (ST11) and was primarily dominated by NDM producers. Within the same node five isolates carrying both KPC and NDM were presented and originated from a single hospital (Hospital A). Although carbapenemases from the OXA-48 family are relatively rare in Bulgaria 12 from the studied 14 OXA-48-like producers were isolated in 2021 from Hospital I and formed a small clonal complex (Figure 2, light blue), which is indicative of a putative outbreak. As hypothesized, all six KVa strains were effectively separated from the remaining KPn strains into separate nodes and formed a clonal complex. They differed by at least three VNTR loci from KPn clusters, while inter-strain distance was smaller. Interestingly, they were isolated in six different hospitals and identified initially as KPn. Correct identification was attained after WGS and were cabapenemases-negative.

### 3.4. Application of MLVA in a Single Hospital

To validate the applicability of MLVA8+ for molecular epidemiology we filtered out KPn strains from set A (*n* = 465) from Hospital A (Figure 5). The subset included 100 strains, collected from four years (2016, 2018, 2019, 2021). Five strains were carbapenemase negative (CP-) and one carried OXA-48. The rest were either NDM (*n* = 26), KPC (*n* = 63) or shared both KPC/NDM (*n* = 5). All CP- were either MT 3 or 27. The five KPC/NDM isolates were either MT2 or 7. Interestingly, all the 2016 KPC strains were MT2 and differed by four-loci from other KPC producers in the following years. With a single exception MT2 strains were not isolated in the following years. The MT3/4 (ST258) were predominantly isolated from ICU, general surgery, cardiac surgery and pediatric surgery, while MT7 (ST11) were found primarily in nephrology, urology, cardiac surgery and general surgery. Interestingly, 37/42 ICU strains were KPC producers mostly isolated from upper respiratory tract and this tendency continued throughout the studied period. In addition, 7/12 isolates from general surgeries were KPC-positive. In contrast, 78.5% of CP+ from urology and nephrology were NDM producers. In cardiac surgical units, both KPC (6) and NDM (5) isolates were present.

## 4. Discussion

A recent study has shown that KpSC comprises of six distinct species that share only 3–4% nucleotide identity, of which KPn, KVa and KQpn [35] are clinically relevant. This division was based on differences in their genetic makeup and evolutionary history [11]. Moreover, WGS revealed extensive sharing of core gene content and plasmid replicons among *Klebsiella* species between strains of both KVa and KQpn producing KPC [35]. According to a KVa population genomics study as close to 80% of all sequenced strains came from sites associated with humans with 40.4% originating from urine and 19% from blood [13], respectively, highlighting the uropathogenicity of KVa. Moreover, it has been suggested that KPn ST258 is a hybrid clone in which 80% of the genome originated from ST11-like strains and 20% from ST442-like strains [36] and MLST is unable to further distinguish it. ST258 strains in both Europe and in the USA are dominant, endemic and associated with high mortality [8,37,38] and resistant to most “last-line” antibiotics, such as amynoglicosides and carbapenems [7]. To address this knowledge gap and the need for an affordable and reliable typing method for accurate subspecies discrimination within the complex, we focused on modifying a multi-locus VNTR analysis method for genotyping KPn, KVa and KQpn.

Initially, the goal was to improve the existing method’s typeability. The original MLVA method by Brink et al. included 224 isolates and was pre-validated on 12 strains. However, for three VNTR loci in two of the validation strains, no alleles could be determined and for 16 more studied strains, at least one VNTR locus dropped out rendering these strains non-typeable. Nevertheless, all these strains were assigned an MT and were included in the phylogenetic analysis. The typeability of the original method was estimated to be 92.85%. In parallel, we wanted to make the method more available by removing the need for costly labelled primers and fragment analysis on Applied Biosystems 3730 sequencer. Therefore, we designed two multiplex PCRs so that each expected amplicon does not overlap with the others within the same reaction. As a result, the typeability increased to 99.78% (1/465 strain, not included). A lack of amplification of some VNTRs was observed occasionally; however, after re-testing, valid bands were obtained. The full typing process requires DNA isolation, PCR setup, PCR program run-time, electrophoretic separation and type assignment (Figure 1) and could be performed in 4–5 h depending on the tested isolates number. In outbreak situations time-to-result is an essential factor to consider. The procedure is affordable, more cost efficient, fairly easy to implement, interpret and can be performed with generic molecular biology laboratory equipment. VNTR allele calling and MLVA type assignment can be conducted with Excel spreadsheet or other more comprehensive software, such as BIONUMERICS.

Initially, we relied entirely on MLST to validate the obtained results. Later on, genomes of clinical strains from a large European study, covering Bulgaria, were included. Genomes with varying repeat copy number were subjected to MLVA8+ and compared to our in silico MLVA typing (see Section 2) for validation purposes. To our surprise, inconsistencies were observed between the VNTR copy number in some of the loci and the in vitro testing. Upon further investigation, it was discovered that the problem was caused by misassembly of short tandem repeats (STRs) using the genome assembly tool MEGAHIT [39]. Although it is a fast and memory-efficient assembly tool, it seemed that STRs regions were misassembled probably due to their repetitive nature and their presence in multiple copies. The errors occurred most frequently in VNTR 27, where the repeats copy number found in Megahit-assembled genomes were always smaller than the actual (data not shown). To solve this problem, we switched to a different genome assembly tool called Unicycler [24], which was able to correctly assemble the STRs and generate results that matched those obtained from in vitro testing. Although repeated regions cause problems for assembly tools, MLVA8+ repeated regions were of no problem due to their short length. The read length of 150 bp for ECDC sequenced and 300 bp for locally sequenced isolates were enough to cover 7/8 VNTR loci hypothetically carrying the highest repeat copy number. For VNTR 10 in all our samples (sequenced or not) the highest observed repeat copy number was 5 (285 bp). This demonstrates the importance of careful validation and the potential impact of choice of genome assembly tool on the accuracy of results in studies involving STR typing methods, such as MLVA.

A major advantage of MLVA8+ is its ability to differentiate and distinguish beyond the ST258 cluster, which was previously challenging using traditional methods, such as multi-locus sequence typing (MLST). ST258 has been associated with the emergence of multi-drug resistant KPn [7,40] and the potential to differentiate between different subspecies within this cluster can help to track the spread and evolution of these strains [41]. This is also important for clinical purposes, as various subspecies of KPn may have different virulence factors and antimicrobial susceptibility profiles. According to the ST258 core-genome SNP phylogeny there are two distinct clades with clade I harboring the KPC-2 carbapenemase gene and the wzi-29 capsular synthesis allele, whereas clade II isolates carried primarily KPC-3 and the wzi-154 capsular synthesis allele. Some ST258 KPC-3 strains were further associated with ceftazidime-avibactam resistance [7], an antimicrobial specifically targeting KPC-producers. It is important here to highlight the relevant implications for the clinical and the scientific community, as it allows for a more detailed understanding of the evolution and spread of antibiotic-resistant strains within these species.

KVa is frequently misidentified as KPn [42,43]. In most cases, in which KVa was the aetiological root-cause of the infection, strains were initially biochemically misidentified as KPn [35,44,45,46]. This could have significant clinical consequences, as KVa has been shown to have specific antimicrobial susceptibility profiles and virulence factors compared to KPn. Recently, a KVa clone carrying acquired NDM-1 and CTX-M-15 in addition to virulence genes typically found in KPn strains were reported [47]. High-risk antibiotic resistance genes were found present in KVa genomes from various regions worldwide and KVa clinical isolates can establish higher bladder titers than KPn due to type 1 pilus and altered *fim* operon, indicating its prominent uropathogenicity [13]. As of today, genome-based methods, such as WGS with SNP typing, and automated methods, such as MALDI-TOF MS (latest libraries only) technique or *KlebSeq*, a sequencing tool for screening and epidemiological surveillance of the *Klebsiella* genus [48], could be effective to correctly identify KVa. However, those methods are either expensive, labor intensive or require costly equipment or expertise. To address this issue, a new MLST scheme for KVa was developed in 2019 [28] and we compared to it the MLVA8+ method. While the Simpson’s indices for both methods were virtually similar, the aWI for congruence was much higher for MLVA8+. This suggests that the MLVA8+ was a more discriminatory method for genotyping KVa than MLST and is a useful tool next to previously mentioned techniques for *Klebsiella* subspecies identification.

KPn ST11 carrying predominantly NDM-1 has been identified as a global threat due to its widespread occurrence and potential to cause outbreaks in hospitals [30,33,49,50]. Similar to MLST, in our sample sets, the MLVA technique effectively and reliably differentiates between different clonal complexes of KPn, including ST11 and can be used to track the hospital spread. To elucidate the epidemiological potential for strain distribution surveillance we tested the MLVA on a large pool of isolated strains collected from a single hospital over a period of 5 years (Figure 5). In 2016, all ICU strains were identified as MT2; however, this type was not observed in later years, as MT3 and MT4 became dominant in 2018, 2019 and 2021. It is possible that over the span of two years the intra-ward persistence was eliminated and a new strain was introduced or MT2 was superseded by the more transmissive MT 3/4 (ST258) [37]. No definitive conclusions could be drawn regarding this possible substitution without complementary genotyping data from 2017 and relevant information on interventions, such as changes to the workflow of the ICU or patient hospitalization history. MT2 was isolated once in 2018, but instead of KPC, it produced NDM. It is nonetheless, noteworthy to elucidate the applicability of MLVA8+ for intra-hospital retrospective investigation of carbapenem-resistant KPn strains to correctly establish phylogenetic relatedness and infer strain transmission among individual wards or longitudinal surveillance in a single unit. Furthermore, OXA-48-like producing strains were reported in hospital I over a short time period, which is indicative of a putative outbreak. After MLVA testing and WGS they were all identified as ST2096 (MLVA type 10) and formed a separate clonal cluster (Figure 2).

A few limitations of the study could be noted. KQpn was reported as a novel species within KpSC in 2014 [51] and is still rarely reported as pathogen mainly due to problems with misidentification. Very few genomes were since published and no dedicated KQpn MLST scheme has been developed. Therefore, the MLVA8+ validation for KQpn was not as extensive as with KVa. Further in vitro validation studies are needed for its wider adoption for KQpn. Next, in two strains BG08S01Kpn (VNTR 53) and BG10S05Kpn (VNTR 51) negative VNTR values were reported. Interestingly, the VNTR 53 amplicon size was an exact multiple of the repeat size, which is compatible with minus four repeats. The VNTR 51 repeat copy number did not vary within the Bulgarian sample set A (*n* = 464), except for BG10S05Kpn (−1 repeat) and it was not multiple of the repeat size, indicating that this value might be an error. Both strains were CP-.

## 5. Conclusions

In this study, we adopted, modified and validated a multi-locus VNTR analysis (MLVA8+) typing method for identifying and characterizing KPn, KVa and, to an extent, KQpn. In a diverse sample set, we found that the updated scheme had an overall discriminatory power of 0.846 and was able to distinguish more accurately isolates with identical STs compared to the classical MLST. A major advantage is the effective differentiation of the KPC carrying hybrid cluster ST258 into two MLVA sub-clusters. Compared to both the original KPn MLST scheme and the novel KVa MLST from 2019, MLVA8+ showed higher discriminatory power (higher adjusted Wallace’ coefficients). The typeability, availability and cost-efficiency of the method improved significantly, while time-to-result was reduced, rendering MLVA8+ suitable for rapid large-scale outbreak investigation, especially for resource-limited laboratories. The applicability of MLVA8+ was demonstrated for intra-hospital retrospective investigation of carbapenem-resistant KPn strains to correctly establish phylogenetic relatedness and infer strain transmission among individual wards or longitudinal surveillance in a single unit. The MLVA8+ method is a viable alternative to other widely used typing techniques for outbreak detection and conducting molecular epidemiological studies due to it providing similar results at lower cost and time.

## Data Availability

All used data is included in the main text and in the Supplementary Materials. Relevant links and/or references to other sources are included in the main text. The generated information and/or datasets analyzed during the current study are available from the corresponding author on reasonable request.

## Supplementary Materials

The following supporting information can be downloaded at: https://www.mdpi.com/article/10.3390/microorganisms11020444/s1, Table S1: Variable Number Tandem Repeats included in the MLVA scheme; Table S2: PCR scheme for MLVA8.1 and MLVA 8.2; Table S3: Comparison of the exact fragment sizes with two reference genomes *Klebsiella quasipneumoniae* ATCC 700603 (yellow) and *Klebsiella pneumoniae* ATCC 13883 (blue) obtained with the original and the modified MLVA8+ scheme; Table S4: In silico convention for the reference genome ATCC 13883 (accession number JOOW00000000); Table S5: All strains (set A); Table S6: KpnMLST vs. MLVA (set B); Table S7: KvMLST (2019) vs. MLVA (set C); Table S8: Calculator for conversion of amplicon size to repeat copy number.
